# Author Correction: Gene-associated markers provide tools for tackling illegal fishing and false eco-certification

**DOI:** 10.1038/s41467-019-13399-5

**Published:** 2019-11-22

**Authors:** Einar E. Nielsen, Alessia Cariani, Eoin Mac Aoidh, Gregory E. Maes, Ilaria Milano, Rob Ogden, Martin Taylor, Jakob Hemmer-Hansen, Massimiliano Babbucci, Luca Bargelloni, Dorte Bekkevold, Eveline Diopere, Leonie Grenfell, Sarah Helyar, Morten T. Limborg, Jann T. Martinsohn, Ross McEwing, Frank Panitz, Tomaso Patarnello, Fausto Tinti, Jeroen K. J. Van Houdt, Filip A. M. Volckaert, Robin S. Waples, FishPopTrace Consortium, Jan E. J. Albin, Juan M. Vieites Baptista, Vladimir Barmintsev, José M. Bautista, Christian Bendixen, Jean-Pascal Bergé, Dietmar Blohm, Barbara Cardazzo, Amalia Diez, Montserrat Espiñeira, Audrey J. Geffen, Elena Gonzalez, Nerea González-Lavín, Ilaria Guarniero, Marc Jerôme, Marc Kochzius, Grigorius Krey, Olivier Mouchel, Enrico Negrisolo, Corrado Piccinetti, Antonio Puyet, Sergey Rastorguev, Jane P. Smith, Massimo Trentini, Véronique Verrez-Bagnis, Alexander Volkov, Antonella Zanzi, Mark de Bruyn, Gary R. Carvalho

**Affiliations:** 1 Section for Population Ecology and Genetics, National Institute of Aquatic Resources, Technical University of Denmark, Vejlsøvej 39, DK-8600 Silkeborg, Denmark; 2 Department of Experimental Evolutionary Biology, University of Bologna, Bologna, I-40126 Italy; 3 Laboratory of Biodiversity and Evolutionary Genomics, Katholieke Universiteit Leuven, Ch. Deberiotstraat, 32, B-3000 Leuven, Belgium; 4 Institute for the Protection and Security of the Citizen (IPSC) Joint Research Centre (JRC) European Commission (EC) JRC.G.4 – Maritime Affairs, Via Enrico Fermi 2749 (TP 051), I-21027 Ispra (Va), Italy; 5 Department of Public Health, Comparative Pathology, and Veterinary Hygiene, University of Padova, viale dell’Università 16, 35020 Legnaro, Italy; 6 TRACE Wildlife Forensics Network, Royal Zoological Society of Scotland, Edinburgh, EH12 6TS UK; 7 Molecular Ecology and Fisheries Genetics Laboratory, School of Biological Sciences, Environment Centre Wales, Bangor University, Bangor, Gwynedd, LL57 2UW UK; 8 Matís, Icelandic Food and Biotech R&D, Vínlandsleið 12, 113, Reykjavík, Iceland; 9 Department of Molecular Biology and Genetics, Faculty of Science and Technology, Aarhus University Blichers Allé 20, DK-8830 Tjele, Denmark; 10 Northwest Fisheries Science Center, National Marine Fisheries Service, National Oceanic and Atmospheric Administration, 2725 Montlake Boulevard East, Seattle, WA 98112-2013 USA; 11 Universidad Complutense de Madrid (UCM), Ciudad Universitaria, 28040 Madrid, Spain; 12 University of Bergen (UiB), Postboks 7800, NO-5020 Bergen, Norway; 13 University of Bremen (UNI.HB), Bibliothekstraße 1, 28359 Bremen, Germany; 14 Département Sciences & Techniques Alimentaires Marines (IFREMER), IFREMER Centre de Nantes, Rue de l’Ile d’Yeu, BP 1105, Nantes, CEDEX 03 France; 15 National Agricultural Research Foundation (NAGREF), Fisheries Research Institute, Nea Peramos, Kavala, GR 64007 Greece; 16 Spanish National Foundation of Fish and Seafood Processors, Area of Molecular Biology and Biotechnology, ANFACO-CECOPESCA, 36310 Vigo, Pontevedra Spain; 17 University of Aarhus (AU), Nordre Ringgade 1, DK-8000 Aarhus C, Denmark; 18 The Centre of Molecular Genetic Identification, Russian Federal Research Institute for Fisheries and Oceanography (VNIRO), 17, V. Krasnoselskaya, 107140 Moscow, Russia; 19 Molecular Ecology, Evolution and Phylogenetics Lab, School of Life and Environmental Sciences, Faculty of Science, The University of Sydney, 2006 NSW, Australia

Correction to: *Nature Communications* 10.1038/ncomms1845, published online 22 May 2012.

The original version of this Article did not acknowledge Mark de Bruyn as part of the FishPopTrace consortium. This has not been corrected in the PDF and HTML versions of the Article.

